# Sphingomyelin is involved in regulating UCP1-mediated nonshivering thermogenesis

**DOI:** 10.1016/j.jlr.2024.100559

**Published:** 2024-05-09

**Authors:** Detian Hu, Houyu Zhang, Zhen Liu, Carlos F. Ibáñez, Cai Tie, Meng Xie

**Affiliations:** 1 Chinese Institute for Brain Research, Zhongguancun Life Science Park, Beijing, China; 2 Academy for Advanced Interdisciplinary Studies, Peking University, Beijing, China; 3 Yuanpei College, Peking University, Beijing, China; 4 School of Life Sciences, Peking University, Beijing, China; 5 Peking-Tsinghua Center for Life Sciences, Beijing, China; 6 PKU-IDG/McGovern Institute for Brain Research, Beijing, China; 7 Department of Neuroscience, Karolinska Institute, Stockholm, Sweden; 8 State Key Laboratory for Fine Exploration and Intelligent Development of Coal Resources, China University of Mining and Technology-Beijing, Beijing, China; 9 School of Chemical and Environmental Engineering, China University of Mining and Technology-Beijing, Beijing, China; 10 School of Psychological and Cognitive Sciences, Beijing Key Laboratory of Behavior and Mental Health, Peking University, Beijing, China; 11 Department of Biosciences and Nutrition, Karolinska Institute, Flemingsberg, Sweden

**Keywords:** sphingomyelin, sphingolipid, adipocyte, adipose tissue, adipogenesis, thermogenesis, UCP1, UPLC-HRMS

## Abstract

Adipogenesis is one of the major mechanisms for adipose tissue expansion, during which spindle-shaped mesenchymal stem cells commit to the fate of adipocyte precursors and differentiate into round-shaped fat-laden adipocytes. Here, we investigated the lipidomic profile dynamics of ex vivo-differentiated brown and white adipocytes derived from the stromal vascular fractions of interscapular brown (iBAT) and inguinal white adipose tissues. We showed that sphingomyelin was specifically enriched in terminally differentiated brown adipocytes, but not white adipocytes. In line with this, freshly isolated adipocytes of iBAT showed higher sphingomyelin content than those of inguinal white adipose tissue. Upon cold exposure, sphingomyelin abundance in iBAT gradually decreased in parallel with reduced sphingomyelin synthase 1 protein levels. Cold-exposed animals treated with an inhibitor of sphingomyelin hydrolases failed to maintain core body temperature and showed reduced oxygen consumption and iBAT UCP1 levels. Conversely, blockade of sphingomyelin synthetic enzymes resulted in enhanced nonshivering thermogenesis, reflected by elevated body temperature and UCP1 levels. Taken together, our results uncovered a relation between sphingomyelin abundance and fine-tuning of UCP1-mediated nonshivering thermogenesis.

Adipose tissue is mostly composed of adipocytes, as well as a variety of other cell types contained in the stromal vascular fraction (SVF), including adipose stem and progenitor cells (ASPCs), immune cells, and vascular cells. Based on morphological and functional differences, adipocytes can be classified into three types, that is white, beige, and brown adipocytes. Brown adipocytes are characterized by the presence of multiple small-sized lipid droplets and a high mitochondria density. Rather than serving as an energy-storing reservoir like the white adipocytes, they are primarily responsible for adaptive nonshivering thermogenesis via the inner mitochondrial membrane anion/H^+^ symporter uncoupling protein 1 (UCP1) (1, 2, 3). When extra heat is needed, sympathetic nerve terminals located in the brown adipose tissue (BAT) release noradrenaline, which, in rodents, interacts with β3-adrenergic receptors to trigger lipolysis via cAMP/protein kinase A signaling (1) and induce UCP1 gene expression via p38 mitogen-activated protein kinase (4). In addition, long-chain fatty acids released through lipolysis directly interact with UCP1 and activate its H^+^ transport activity for heat production (3). Beige adipocytes located in subcutaneous fat depots resemble brown adipocytes in possessing inducible and reversible thermogenic capacity in response to reduced ambient temperature; however, similar to white adipocytes, they show very low UCP1 expression levels at thermo-neutral temperatures (5, 6).

Lipids are one of the four major molecular constituents of biological organisms and play important roles in many essential cellular processes, including cell signaling, energy storage, and serving as building blocks for cellular membranes. The LIPID MAPS Consortium (7) classified lipids into eight categories, namely, fatty acyls, glycerolipids, glycerophospholipids, sphingolipids, sterol lipids, prenol lipids, saccharolipids, and polyketides. Lipid composition varies substantially among different cell types and is tightly correlated with the regulation of cellular function and homeostasis (8). In primary white, beige, and brown adipocytes, lipidome comparisons have revealed a correlation between the profile of ceramide species and induction of thermogenic capacity, also known as “browning” (9). Exposure to acute cold leads to a dramatic remodeling of triglyceride and glycerophospholipid depots in BAT, in parallel with changes in the expression of genes encoding lipid-synthesizing enzymes (10).

Sphingolipids, including ceramide, sphingomyelin, and glycosphingolipid, constitute a diverse class of cellular lipids that carry out essential functions in cell signaling, cell-cell recognition, and membrane structure stabilization (11). Structurally, all sphingolipids contain a long-chain aliphatic amine backbone, known as sphingoid base. Addition of an amide-linked fatty acid chain with various lengths to the backbone constitutes a ceramide molecule, which can be further derivatized into sphingomyelin or glycosphingolipid by the addition of a phosphocholine or saccharide headgroup(s), respectively (12). Sphingomyelins are localized in membrane rafts and caveolae, where they play important roles in cell signaling and membrane trafficking of lipids and proteins (13). A reference map of sphingolipid distribution in twenty-one murine tissues has uncovered the presence of high levels of sphingomyelin in BAT of female mice (14). Sphingomyelin synthases (SMS) catalyze the synthesis of sphingomyelin by transferring a phosphocholine molecule from phosphatidylcholine to a ceramide molecule (15). Among the three isoforms of SMS, SMS1 resides in the Golgi apparatus and is mainly responsible for the cellular sphingomyelin production, while SMS2 is located in the plasma membrane that catalyzes the reversible conversion of ceramide to sphingomyelin (16). On the other hand, hydrolysis of sphingomyelin into ceramide and phosphocholine is catalyzed by the sphingomyelin phosphodiesterases (SMPDs), including neutral (SMPD2, 3, and 4) and acidic types (SMPD1) (17).

The dynamics of lipid species abundance during adipogenesis (the process by which ASPCs differentiate into mature adipocytes) of brown and white adipocytes has not been investigated. In the present study, we examined temporal changes in global lipidomic profiles in primary cultures of ASPCs isolated from interscapular BAT (iBAT) and inguinal white adipose tissue (iWAT) as they differentiated into mature brown and white adipocytes, respectively. We found sphingomyelins to be specifically accumulated during differentiation of brown adipocytes. Functional studies further revealed that these lipids play important roles in the regulation of UCP1-mediated nonshivering thermogenesis.

## Materials and methods

### Mice

All mice experiments were performed in accordance to the protocol approved by the Institutional Animal Care and Use Committee of Peking University (Psych-XieM-2) and the Chinese Institute of Brain Research (CIBR-IACUC-035). Mice were housed in specific pathogen free condition under a 12:12 h light/dark cycle with free access to food and water. All animals used in the present study were C57BL/6J male mice. For cold exposure, animals were housed individually in a temperature and light-controlled incubator (Darth Carter, Hefei, China). Rectal temperature was measured with a BAT-12 microprobe thermometer (Physitemp). D609 (Macklin, D877166) was dissolved in double distilled water and injected intraperitoneally into the mice at a concentration of 50 mg/kg. GW4869 (Macklin, G873076) was dissolved in 10% DMSO and injected intraperitoneally into the mice at a concentration of 2.5 mg/kg.

### Mouse SVF cell isolation and primary culture for adipogenic differentiation

4 to 6-week-old C57BL/6J male mice were used for SVF isolation. iBAT and iWAT were dissected and washed twice with Hank’s balanced salt solution, before mincing into small pieces with scissors. Minced tissues were digested with 1 mg/ml collagenase type II (Gibco, EC0111) dissolved in digestion buffer containing 0.1 M Hepes sodium salt (Solarbio, H8090), 0.12 M sodium chloride (Solarbio, S8210), 50 mM potassium chloride (Solarbio, P9921), 5 mM d-glucose (Solarbio, G8150), 1 mM calcium chloride (Solarbio, C7250), and 1.5% bovine serum albumin (Solarbio, A8010) with constant shaking at 300 rpm at 37°C for 60 min. An equal volume of DMEM/F12 (Gibco, 11330032) supplemented with 10% fetal bovine serum (Gibco, 10500), 2 mM Glutamax (Gibco, 35050061), 0.1 mM nonessential amino acids (Gibco, 11140050), and 100 U/ml penicillin-streptomycin (growth media) was added to the mixture at the end of digestion. iBAT samples were filtered through 70-μm cell strainers and subsequently centrifuged at 400 rcf for 10 min to separate floating adipocytes. iWAT samples were filtered through 100-μm cell strainers followed by centrifuging at 450 rcf for 10 min. After removing the adipocytes and supernatant, the pellet was resuspended in 2 ml red cell lysis buffer (Solarbio, R1010) and incubated at 37°C for 5 min to remove blood cells. An equal volume of growth media was added after the incubation. iBAT samples were directly centrifuged at 400 rcf for 5 min. iWAT samples were filtered through 20-μm cell strainers before centrifugation. The resulting pellet was washed with DMEM/F12, supplemented with 5% fetal bovine serum and 100 U/ml penicillin-streptomycin (basal medium), before plating in gelatin (0.1%, Sigma–Aldrich, G9391)-coated plates for growth in growth media.

For adipogenic induction of iBAT SVF cells, DMEM/F12 supplemented with 5% heat-inactivated fetal bovine serum, 125 μM indomethacin (Sigma-Aldrich, I7378), 15 mM Hepes (Sigma-Aldrich, H4034), 33 μM d-biotin (Sigma-Aldrich, B4639), 17 μM pantothenate (Sigma-Aldrich, P5155), 0.5 mM 3-isobutyl-1-methylxanthine (Sigma-Aldrich, I5879), 1 μM dexamethasone (Sigma-Aldrich, D4902), 2.5 μM rosiglitazone (Sigma-Aldrich, R2408), 2 nM 3,3’,5-Triiodo-L-thyronine sodium salt (Sigma-Aldrich, T6397), 1 μg/ml insulin (Sigma-Aldrich, I3536), 0.55 μg/ml transferrin (Sigma-Aldrich, T0665), 0.67 ng/ml sodium selenite (Invitrogen, 41400045), 2 mM Glutamax, 0.1 mM nonessential amino acids, and 100 U/ml penicillin-streptomycin was applied to the culture for 3 days. For adipogenic induction of iWAT SVF cells, DMEM/F12 supplemented with 5% heat-inactivated fetal bovine serum, 33 μM d-biotin, 17 μM pantothenate, 0.5 mM 3-isobutyl-1-methylxanthine, 1 μM dexamethasone, 1 μM rosiglitazone, 2 nM 3,3’,5-Triiodo-L-thyronine sodium salt, 1 μg/ml insulin, 0.55 μg/ml transferrin, 0.67 ng/ml sodium selenite, 2 mM Glutamax, 0.1 mM nonessential amino acids, and 100 U/ml penicillin-streptomycin was applied to the culture for 3 days. After induction, iBAT cells were maintained in DMEM/F12 supplemented with 5% heat-inactivated fetal bovine serum, 2.5 μM rosiglitazone, 33 μM d-biotin, 17 μM pantothenate, 1 μM dexamethasone, 10 μg/ml insulin, 2 mM Glutamax, 0.1 mM nonessential amino acids, and 100 U/ml penicillin-streptomycin until full differentiation. iWAT cells were maintained in DMEM/F12 supplemented with 5% heat-inactivated fetal bovine serum, 33 μM d-biotin, 17 μM pantothenate, 1 μM dexamethasone, 10 μg/ml insulin, 2 mM Glutamax, 0.1 mM nonessential amino acids, and 100 U/ml penicillin-streptomycin. Medium was changed every 2 days during the period of differentiation and maintenance.

For chemical treatment of fully differentiated brown adipocytes on days in vitro (DIV)9, 100 μM D609, 20 μM GW4869, 10 μM CL, and a protease inhibitor cocktail stock solution (MCE, HY-K0010) diluted in 1:200 ratio were applied to the culture for 12, 6, 12, and 12 h, respectively. For chemical treatment of primary cells on DIV4, 100 μM D609 and 20 μM GW4869 were applied to the culture for 12 and 6 h, respectively.

### Oil Red O staining of ex vivo-differentiated adipocytes

Oil Red O stock solution was prepared by dissolving 0.7 g Oil Red O powder in 200 ml isopropanol and filtered through a 0.2-μm filter. Oil Red O working solution was prepared by 60% dilution of the stock in distilled water. Cells were fixed in 10% paraformaldehyde for 1 h, before being stained in the Oil Red O working solution for 1 h with gentle shake. Images were taken by a Zeiss Axio Observer microscope.

### Lipidomic analysis

Lipids were extracted from 300 μl volume of cells, 50 mg adipose tissue or adipocytes, and SVF extracted from 100 mg adipose tissue with 1 ml of MTBE/MeOH (10/1, v/v). The lipid-containing upper layer was dried with vacuum concentrator and stored at −20°C before re-dissolving in MeOH for analysis. The bottom layer was used for protein quantification using a BCA protein assay kit (Solarbio, PC0020). All lipid concentration presented in the study was normalized to the protein concentration of the same sample and was presented as μg lipid per mg protein.

Untargeted lipidomic analysis was performed on Waters ultra-performance liquid chromatography I-Class SYNAPT G2-Si Mass Spectrometer (Waters). Waters ACQUITY ultra-performance liquid chromatography BEH C8 (2.1 × 100 mm) column (Waters) was used for chromatographic separation. Column temperature was kept at 40°C. Injection volume was 2 μl per sample. Mobile phase A consisted of 10 mM ammonium acetate in acetonitrile (ACN)/water (3/2, v/v), supplemented with 0.1% formic acid. Mobile phase B consisted of 10 mM ammonium acetate in ACN/isopropyl alcohol (1/9, v/v), supplemented with 0.1% formic acid. Elution condition was as follows: 80% mobile phase A and 20% mobile phase B at 0 min, 75% mobile phase A and 25% mobile phase B at 2 min, 70% mobile phase A and 30% mobile phase B at 2.1 min, 65% mobile phase A and 35% mobile phase B at 12 min, 30% mobile phase A and 70% mobile phase B at 12.1 min, 1% mobile phase A and 99% mobile phase B at 18 min, 80% mobile phase A and 20% mobile phase B at 18.1 min, 80% mobile phase A and 20% mobile phase B at 20 min. The mode of detection was full scan in positive-mode. Internal standard used for quantification was SPLASH® II LIPIDOMIX® Mass Spec Standard (Avanti Polar Lipids), containing 15:0-18:1(d7) phosphatidylcholine, 15:0-18:1(d7) phosphatidylethanolamine, 18:1(d7) lyso-phosphatidyl-choline, 18:1(d7) Chol Ester, C18(Plasm)-18:1(d9)phosphatidylcholine, 15:0-18:1(d7) diglyceride, 15:0-18:1(d7)-15:0 triglyceride, and d18:1-18:1(d9) sphingomyelin (SM).

Sphingolipid analysis was performed on Dionex U3000-SCIEX 5500 QTRAP with electrospray ionization source (SCIEX). Waters ACQUITY ultra-performance liquid chromatography BEH C8 (2.1×100 mm) column was used for chromatographic separation (Waters). Column temperature was kept at 60°C. Injection volume was 5 μl per sample. Mobile phase A consisted of 10 mM ammonium acetate in water, supplemented with 0.1% formic acid. Mobile phase B consisted of 10 mM ammonium acetate in ACN/isopropyl alcohol (4/3, v/v), supplemented with 0.1% formic acid. Elution condition was as follows: 30% mobile phase A and 70% mobile phase B at 0 min, 1% mobile phase A and 99% mobile phase B at 5 and 10 min, 30% mobile phase A and 70% mobile phase B at 10.5 and 15 min. The mode of detection was multiple reaction monitoring in positive-mode. Internal standard used for quantification was CER Internal Standard Mixture - UltimateSPLASH™ (Avanti Polar Lipids), containing C16:1 Ceramide-d7 (d18:1-d7/16:1), C18:1 Ceramide-d7 (d18:1-d7/18:1), C20:1 Ceramide-d7 (d18:1-d7/20:1), C22:1 Ceramide-d7 (d18:1-d7/22:1), and C24:1 Ceramide-d7 (d18:1-d7/24:1).

For galactosylceramide and glucosylceramide analysis, 50 mg of tissue were extracted with 1 ml of organic solvent (MTBE/MeOH, 10/1, v/v). After vortex, the supernatant was concentrated at 45°C and re-dissolved in 100 μl ACN for solid-phase extraction (SPE). SPE was processed on Oasis HLB 96-Well Plates (Waters) with MeOH and water as activating and washing solvent for SPE cartridges, respectively. MeOH/water (5/95, v/v) was used as washing solvent for samples. Galactosylceramide and glucosylceramide were eluted by MeOH/ACN (90:10, v/v). The eluent was concentrated at 45°C and the dried samples were stored at −20°C until redissolution. Internal standard used to quantify target substance was C22 glucosylceramide-d4 (d18:1/22:0-d4) (Avanti Polar Lipids). Three standards used for target substance characterization were glucosylceramide (Soy), C16 galactosyl(β) ceramide (d18:1/16:0), and C16 glucosyl(β) ceramide (d18:1/16:0) (Avanti Polar Lipids). The remnant of supernatant was used for protein quantification with BCA protein assay kit (Solarbio). The concentration of galactosylceramide and glucosylceramide was normalized to protein concentration. The analysis of target substance was performed on Waters UPLC I-Class Synapt G2-Si. Halo HILIC (4.6 × 150 mm) column was employed for chromatographic separation. The column temperature was controlled at 30°C. The injection volume was 5 μl. The flow rate was 0.5 ml/min. Mobile phase consisted of 5 mM ammonium formate in ACN/MeOH/water (38/1/1) with 0.5% formic acid. Elution condition was isocratic elution. The mode of detection was full scan in positive-mode.

All identified compound was mapped to the LIPID MAPS database (7) at the “category,” “main class,” “subclass,” and “abbrev” levels. Features that matched the same lipid ID were merged. Data analysis was performed with the MetaboAnalystR package (version 3.1.0) (18). Principal component analysis was conducted using the “PCA.Anal” function. Differential lipids were determined by the “ANOVA” methods through the “ANOVA.Anal” function and presented in the form of heatmap using the “PlotSubHeatMap” function. All analyses can be reproduced through custom codes deposited in Github (https://github.com/HouyuZhang/Lipidomics_analysis).

### Immunoblotting

Cells were washed twice with cold PBS and lysed within the wells with RIPA buffer (Sigma–Aldrich, R0278), supplemented with 1% protease inhibitor cocktail (MCE, HY-K0010), 1% phosphatase inhibitor cocktail II (MCE, HY-K0022), and 1% phosphatase inhibitor cocktail III (MCE, HY-K0023). Adipose tissues were minced in RIPA buffer and centrifuged at 12,000 rpm for 5 min. Supernatant was used for protein extraction. Total protein concentration was measured using the BCA protein assay kit. Equal amounts of protein were loaded. Electrophoresis was performed to resolve proteins based on their molecular weight. Resolved proteins were electrotransferred onto polyvinylidene difluoride membrane (Bio-Rad) using a Trans-Blot Turbo Transfer System (Bio-Rad) at 25 V. Membranes were blocked with 3% Blotting-Grade Blocker (Bio-Rad, 1706404) in 1× Tris-buffered saline supplemented with 0.1% Tween-20 (TBST) for 1 h. The following primary antibodies were used for overnight incubation at 4°C in 1% Blotting-Grade Blocker in 1× TBST: UCP1 (Sigma-Aldrich, U6382), α-Tubulin (Proteintech, 11224-1-AP), SMS1 (Abcam, ab235057), SMS2 (LS Bio, LS-C169045), SMPD2 (Abcam, ab131330). Membranes were washed 3 times with 1× TBST and incubated with horseradish peroxidase-conjugated anti-rabbit IgG secondary antibody (Cell Signaling, 7074) in 1% Blotting-Grade Blocker in 1× TBST. Membranes were imaged with the ImageQuant 800 system (Amersham). Band intensity was quantified using the ImageQuant TL software (https://www.cytivalifesciences.com/en/us/shop/protein-analysis/molecular-imaging-for-proteins/imaging-software/imagequant-tl-10-2-analysis-software-p-28619).

### RNA isolation and quantitative RT-PCR analysis

Adipose tissues and cells were homogenized in TRIzol reagent (Invitrogen) for RNA extraction. RNA concentration and purity were assessed using a NanoDrop One spectrophotometer (Thermo Fisher Scientific). Total RNA was reverse transcribed using the RevertAid First Strand cDNA Synthesis Kit (Thermo Fisher Scientific, K1622). cDNA equivalent to 2 ng RNA was used for amplification in 96-well plates in a Cytation 5 Cell Imaging Multimode Reader (BioTek) in a final reaction volume of 10 μl, containing 5 μl PowerUP SYBR Green master mix (AppliedBiosySDs) and 1 μM primer mix (Rui Biotech). All samples were performed in triplicate. Cycle threshold (Ct) values for individual reactions were determined using the CFX Manager (Bio-Rad). For data analysis, Ct-value of gene of interest was first normalized against the housekeeping gene Ct-value by the following equation: ΔCt (Gene) = Ct (Gene) - Ct (Ywhaz). Relative mRNA level was calculated as 2ˆΔCt (Gene). Relative fold change for mRNA level was calculated as 2ˆΔΔCt (Gene), in which ΔΔCt (Gene) = ΔCt (Gene) of samples from different experimental condition – average of ΔCt (Gene) of controls. Primer sequences used are as follows:

Ucp1_forward GGCCTCTACGACTCAGTCCA

Ucp1_reverse TAAGCCGGCTGAGATCTTGT

Cideα_forward TGCTCTTCTGTATCGCCCAGT

Cideα_reverse GCCGTGTTAAGGAATCTGCTG

Pgc1α_forward AGCCGTGACCACTGACAACGAG

Pgc1α_reverse GCTGCATGGTTCTGAGTGCTAAG

Pparγ_forward TCGCTGATGCACTGCCTATG

Pparγ_reverse GAGAGGTCCACAGAGCTGATT

Leptin_forward GAGACCCCTGTGTCGGTTC

Leptin_reverse CTGCGTGTGTGAAATGTCATTG

Adipoq_forward GAAGCCGCTTATGTGTATCGC

Adipoq_reverse GAATGGGTACATTGGGAACAGT

Ywhaz_forward CAGTAGATGGAGAAAGATTTGC

Ywhaz_reverse GGGACAATTAGGGAAGTAAGT

### Whole-body metabolic status assessment

Indirect calorimetry was assessed using a comprehensive laboratory animal monitoring system (Columbus Instruments, Columbus, OH). Mice were housed individually with free access to food and water during the experimental period. Mice were acclimatized to the metabolic cages and kept at 4°C for 3 days before injection with vehicle or GW4869, followed by a 24-h period of automated recording.

### Transmission electron microscopy

iBAT kept under room temperature and cold exposure conditions were fixed with 2.5% (v/v) glutaraldehyde dissolved in 0.1 M phosphate buffer (50 mM Na_2_HPO_4_, 50 mM NaH_2_PO_4_, pH 7.4) for 2 h at room temperature, before infiltrating in 0.1 mol/L imidazole for 1 h. Samples were then postfixed with 1% (wt/vol) osmium tetroxide and 0.1 mol/L imidazole for 1 h. After several washes with distilled water, samples were stained in 1% uranyl acetate at 4°C overnight. Samples were washed in distilled water after the staining and were subsequently dehydrated in a graded ethanol series before embedding in the Embed 812 resin kit (Electron Microscopy Sciences). Samples were cut into 70 nm sections using an ultramicrotome (Leica Microsystem, UC7) and were subsequently collected on single-slot copper grids before staining with uranyl acetate and lead citrate. Tissue sections were imaged using a JEM-1400Flash electron microscope (JEOL) equipped with a 20-megapixel XAROSA digital camera (EMSIS).

### Immunostaining

iBAT was fixed in 1% paraformaldehyde at room temperature for 1 h right after dissection. The tissue was subsequently sectioned into 300-μm-thick slices using a vibratome (Leica, VT1200). Tissue sections were incubated with antibody against Perilipin (Cell Signaling, #9349) at 4°C overnight, followed by 1-h incubation with an anti-rabbit secondary antibody conjugated with Alexa Fluor 555 (Thermo Fisher Scientific, A-31572) at room temperature. Images were taken with an Airyscan 2 LSM 900 confocal microscope (ZEISS).

### Statistics

Statistical analyses were performed using GraphPad Prism version 10.0.2 (https://www.graphpad.com/updates/prism-10-2-0-release-notes). Results were presented as mean ± standard deviation in all figures. Unpaired *t* test with Welch's correction, one-way ANOVA Tukey’s multiple comparisons test, one-way ANOVA Tukey’s post-hoc test, and two-way ANOVA were used to test statistical significance as specified in the corresponding figure legends. The level of statistical significance was assigned as ∗ for *P* < 0.05, ∗∗ for *P* < 0.01, ∗∗∗ for *P* < 0.001, ∗∗∗∗ for *P* < 0.0001. For nonsignificant comparisons, *P* values less than 0.2 were indicated in the graph.

## Results

### Lipidomic dynamics during ex vivo differentiation of brown and white adipocytes

**Fig. 1:**
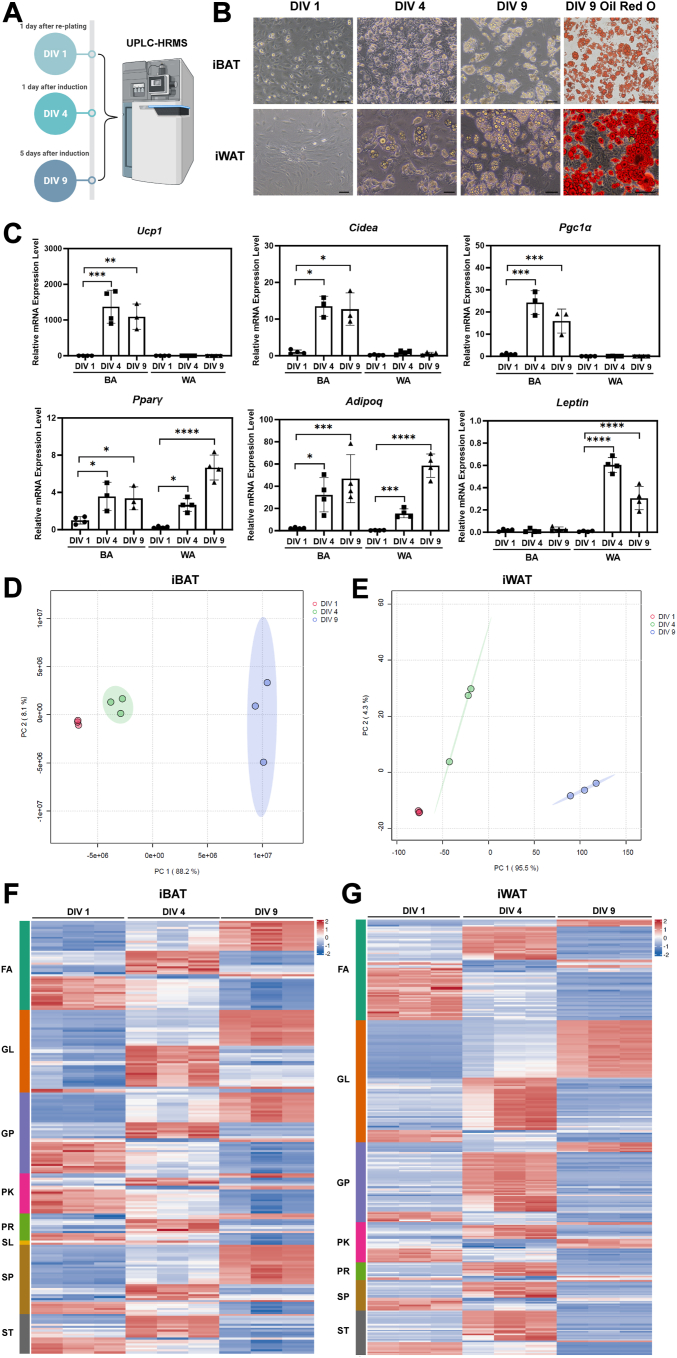
Lipidomic dynamics during ex vivo brown and white adipocyte differentiation. A: Schematic illustration of the collection time points along the differentiation time line. Cells were collected on DIV 1 (1 day after re-plating), DIV 4 (1 day after addition of induction medium), and DIV 9 (fully differentiated adipocytes) for untargeted UPLC-HRMS analysis. B: Representative images of primarily cultured iBAT and iWAT SVF cells on DIV 1, 4, and 9, and Oil Red O staining of derived adipocytes on DIV 9. Scale bar = 100 μm. C: qPCR analysis of *Ucp1*, *Cidea*, *Pgc1α*, *Pparγ*, *AdipoQ*, and *Lep* mRNA levels in iBAT and iWAT SVF primary cultures. n = 3–4 independent experiments, represented by a dot in the graph. ∗*P* < 0.05, ∗∗*P* < 0.01, ∗∗∗*P* < 0.001, ∗∗∗∗*P* < 0.0001 by one-way ANOVA Tukey’s multiple comparisons test. (D, E) Principal component analysis of integrated lipidomic datasets of iBAT (D) and iWAT (E) SVF primary cultures. n = 3 independent experiments, represented by a dot in the graph. (F, G) Heatmap illustrating the dynamics in abundance of the eight major lipid categories in iBAT (F) and iWAT (G) SVF primary cultures. Z-score was used for data presentation. BA, brown adipocyte; DIV, days in vitro; FA, fatty acyl; GL, glycerolipid; GP, glycerophospholipid; iBAT, interscapular brown adipose tissue; iWAT, inguinal white adipose tissue; PK, polyketide; PR, prenol lipid; WA, white adipocyte; SL, saccharolipid; SP, sphingolipid; ST, sterol lipid; SVF, stromal vascular fraction; UCP1, uncoupling protein 1; UPLC-HRMS, ultra-performance liquid chromatography combined with high resolution mass spectrometry.

We investigated the dynamics of lipidomic profiles during ASPC differentiation to brown and white adipocytes in the cultures of primary SVF cells isolated from iBAT and iWAT of 4- to 6-week-old C57BL/6J male mice. Cells were collected after 1, 4, and 9 DIV for untargeted lipidomic analysis that unbiasedly screens all lipids in the samples using ultra-performance liquid chromatography combined with high resolution mass spectrometry (UPLC-HRMS) (Fig. 1A). As the differentiation process proceeded, the spindle-shaped ASPCs gradually became round-shaped adipocytes that were filled with Oil Red O-stained lipids (Fig. 1B). Molecularly, an increasing trend in the expression levels of brown (*Ucp1*, *Cidea*, *Pgc1α*), white (*Lep*), and pan (*Pparγ*, *Adipoq*) adipocyte markers was observed in iBAT and iWAT SVF primary cultures, respectively (Fig. 1C). In the integrated lipidomic datasets, a total of 944 and 1,250 lipid features were identified for the two cultures, respectively. Principal component analysis revealed that the lipid profiles of cells from the three differentiation time points were well separated, indicating distinct and dynamic lipidomic signatures during ex vivo adipogenesis (Fig. 1D, E). Most of the glycerolipid species reached their highest abundance on either DIV 4 or DIV 9 for both brown and white adipocytes (Fig. 1F, G), as expected from cells with enlarged lipid storage. Abundance of fatty acyl and glycerophospholipid species was rather evenly distributed among the three differentiation stages of brown adipocytes (Fig. 1F), whereas fatty acyl species were mostly accumulated on either DIV 1 or DIV 4 during white adipocyte differentiation and most of the glycerophospholipid species were most abundant on DIV 4 (Fig. 1G). The most striking difference in lipidomic profiles between brown and white adipocyte differentiation was observed in the sphingolipid category. Majority of the sphingolipid species were most abundant in fully differentiated brown adipocytes on DIV 9 (Fig. 1F). In contrast, sphingolipids were accumulated in either the precursor (DIV 1) or the intermediate stage (DIV 4) of white adipocyte differentiation (Fig. 1G).

### Sphingomyelins were specifically accumulated in brown adipocytes

**Fig. 2:**
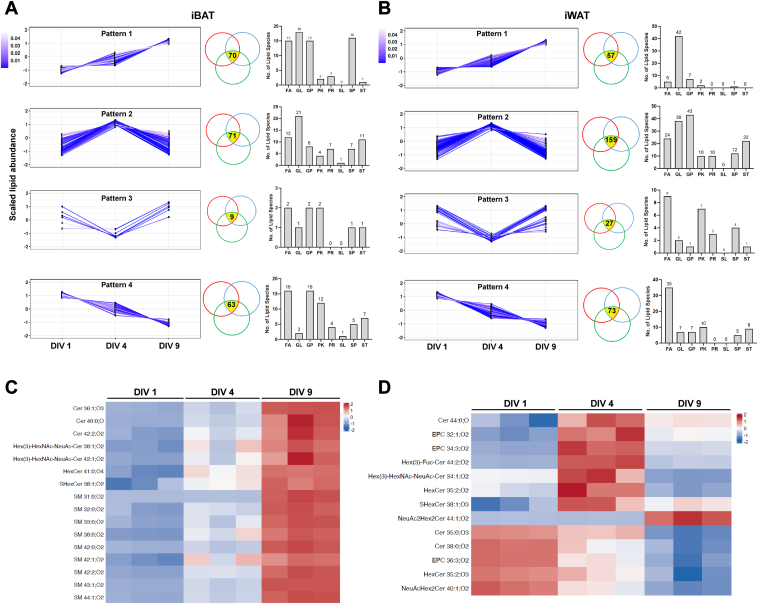
Sphingomyelins were specifically accumulated along the ex vivo brown adipocyte differentiation timeline. A, B: Changing patterns of lipid features, whose abundance were significantly altered in all comparisons between each of the two differentiation time points of iBAT (A) and iWAT (B) SVF primary cultures. Z-score was used for data presentation. Each line represented one lipid feature. Each circle of the Venn diagrams represented one pairwise comparison: *red*, DIV 1 versus DIV 4; *blue*, DIV 4 versus DIV 9; *green*, DIV 1 versus DIV 9. The number of lipid species that was significantly different among all three comparisons was indicated in the overlapped area colored in *yellow* in the Venn diagrams. The distribution of lipid categories in each pattern was plotted in the histogram. n = 3 independent experiments. C: Sphingolipids that were enriched in differentiated brown adipocytes were mostly sphingomyelins. Z-score was used for data presentation. D: Several species of ceramide and hexosylceramide exhibited decreasing trend in abundance during the brown adipocyte differentiation process. Cer, ceramide; DIV, days in vitro; EPC, ethanolamine phosphorylceramide; FA, fatty acyl; GL, glycerolipid; GP, glycerophospholipid; HexCer, hexosylceramide; iBAT, interscapular brown adipose tissue; iWAT, inguinal white adipose tissue; SVF, stromal vascular fraction; PK, polyketide; PR, prenol lipid; SL, saccharolipid; SM, sphingomyelin; SP, sphingolipid; ST, sterol lipid.

To identify the lipid species that exhibited the most dramatic changes during brown and white adipogenesis, we performed pairwise comparisons between cells of each of the two stages and found a total of 235 and 231 lipid features that were significantly different in all comparisons, respectively. Four types of dynamic patterns were identified: lipids that showed either an increasing (Pattern 1) or decreasing (Pattern 2) trend in abundance during differentiation and lipids that had either the highest (Pattern 3) or the lowest (Pattern 4) abundance at the intermediate stage (DIV 4) (Fig. 2A, B). For the subsequent analysis, we focused on Pattern 1 lipids, since they were the most abundant in fully differentiated brown and white adipocytes, indicating their more significant roles in those cells. The majority of pattern 1 lipids in white adipocytes were glycerolipids, which is in line with the gradual accumulation of energy storage lipids in these cells (Fig. 2B). In contrast, pattern 1 lipids in brown adipocytes were mainly composed of a comparable portion of fatty acyls, glycerolipids, glycerophospholipids, and sphingolipids (Fig. 2A). Interestingly, more than half of pattern 1 sphingolipid species were sphingomyelins (Fig. 2C). On the other hand, pattern 4 sphingolipids which exhibited a decreasing trend during brown adipocyte differentiation were mainly ceramides and hexosylceramides (Fig. 2D). These results suggest that sphingomyelins may have distinct functions in fully differentiated brown adipocytes.

**Fig. 3:**
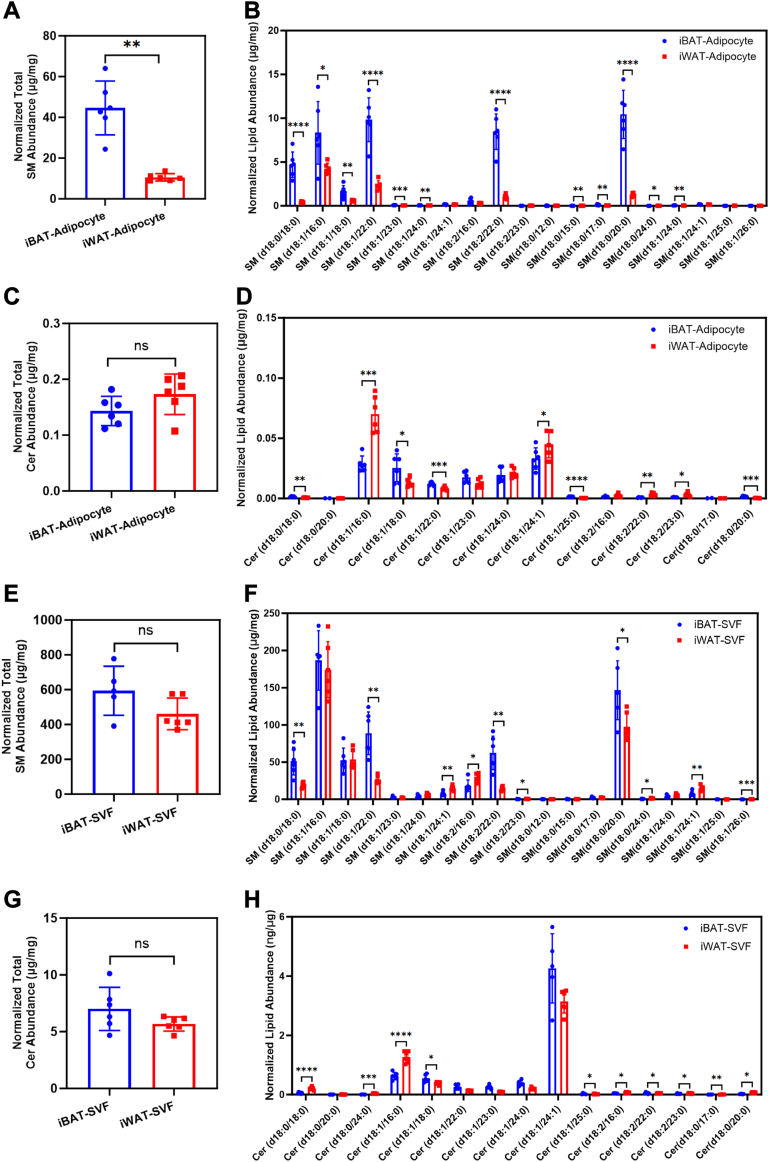
Freshly isolated adipocytes from iBAT contained more sphingomyelins than iWAT. A–D: Adipocytes of iBAT had higher sphingomyelin (A, B), but not ceramide (C, D) abundance, than that of iWAT. n = 5–6 animals per condition, represented by a dot in the graphs. ∗*P* < 0.05, ∗∗*P* < 0.01, ∗∗∗*P* < 0.001, ∗∗∗∗*P* < 0.0001 by unpaired *t* test with Welch's correction. E–H: SVFs of iBAT and iWAT contained comparable levels of sphingomyelins (E, F) and ceramides (G, H). n = 4–6 animals per condition, represented by a dot in the graphs. iBAT, interscapular brown adipose tissue; iWAT, inguinal white adipose tissue.

Next, we used targeted UPLC-HRMS with higher sensitivity that can distinguish the length and degree of saturation of the sphingosine and fatty acid chains in the sphingomyelin molecule to assess the quantitative changes of the sphingolipid profiles of iBAT and iWAT in 10-week-old C57BL/6J male mice. In isolated adipocytes, total sphingomyelin abundance (Fig. 3A) and many individual sphingomyelin species (Fig. 3B) were significantly more abundant in iBAT than iWAT. Next, we intended to link these sphingomyelin species to the ones that were identified by untargeted UPLC-HRMS (Fig. 2C). Since the untargeted UPLC-HRMS cannot distinguish the sphingosine and fatty acid chains of the sphingomyelin molecule, we could only add the number of carbon atom and double bond for the sphingosine and fatty acid chains of each sphingomyelin species identified by targeted UPLC-HRMS to estimate their resemblance to the untargeted data. In this way, we found 5 matched species to the pattern 1 sphingomyelin (Fig. 2C), that is, SM 33:0 (SM 18:0/15:0), SM 38:0 (SM 18:0/20:0), SM 42:0 (SM 18:0/24:0), SM 42:1 (SM 18:1/24:0), and SM 42:2 (SM 18:1/24:1), all of which were significantly higher in iBAT adipocyte, except SM 42:2 (SM 18:1/24:1). In contrast, total ceramide abundance was comparable between the two tissues (Fig. 3C) and individual ceramide species did not exhibit a consistent trend in their relative abundance (Fig. 3D). Notably, sphingomyelin and ceramide content in SVFs of either tissue did not show any consistent pattern (Fig. 3E–H). These results are in line with our ex vivo lipidomic analysis, which further supports the functional importance of sphingomyelin content in brown adipocytes.

### Cold exposure reduced sphingomyelin abundance and SMS1 expression in iBAT

Next, we wondered whether iBAT sphingomyelin levels are affected by cold exposure. As the onset of nonshivering thermogenesis is tightly correlated with increase in UCP1 protein levels in BAT (19), we first set out to determine the precise timing of nonshivering thermogenesis onset after transferring animals from room temperature to cold chambers. For this purpose, we collected iBAT and iWAT from 10-week-old mice housed at 4°C for 16, 24, 40, and 72 h and assessed UCP1 levels. As expected, UCP1 protein level increased progressively with the length of cold exposure and a statistical significance could be observed from 40 h onwards (supplemental Fig. S1A–C). *Ucp1* mRNA level showed a significant increase already after 16-h cold exposure (supplemental Fig. S1D). It is worth mentioning that our mice are routinely housed at a room temperature of 22°C that is lower than thermoneutrality, suggesting that BAT is already partially recruited in our mice under normal housing conditions. For iWAT, due to the initially very low basal level of UCP1 protein in the tissue, cold exposure for 16 h was sufficient to induce a significant increase (supplemental Fig. S1E, F).

**Fig. 4:**
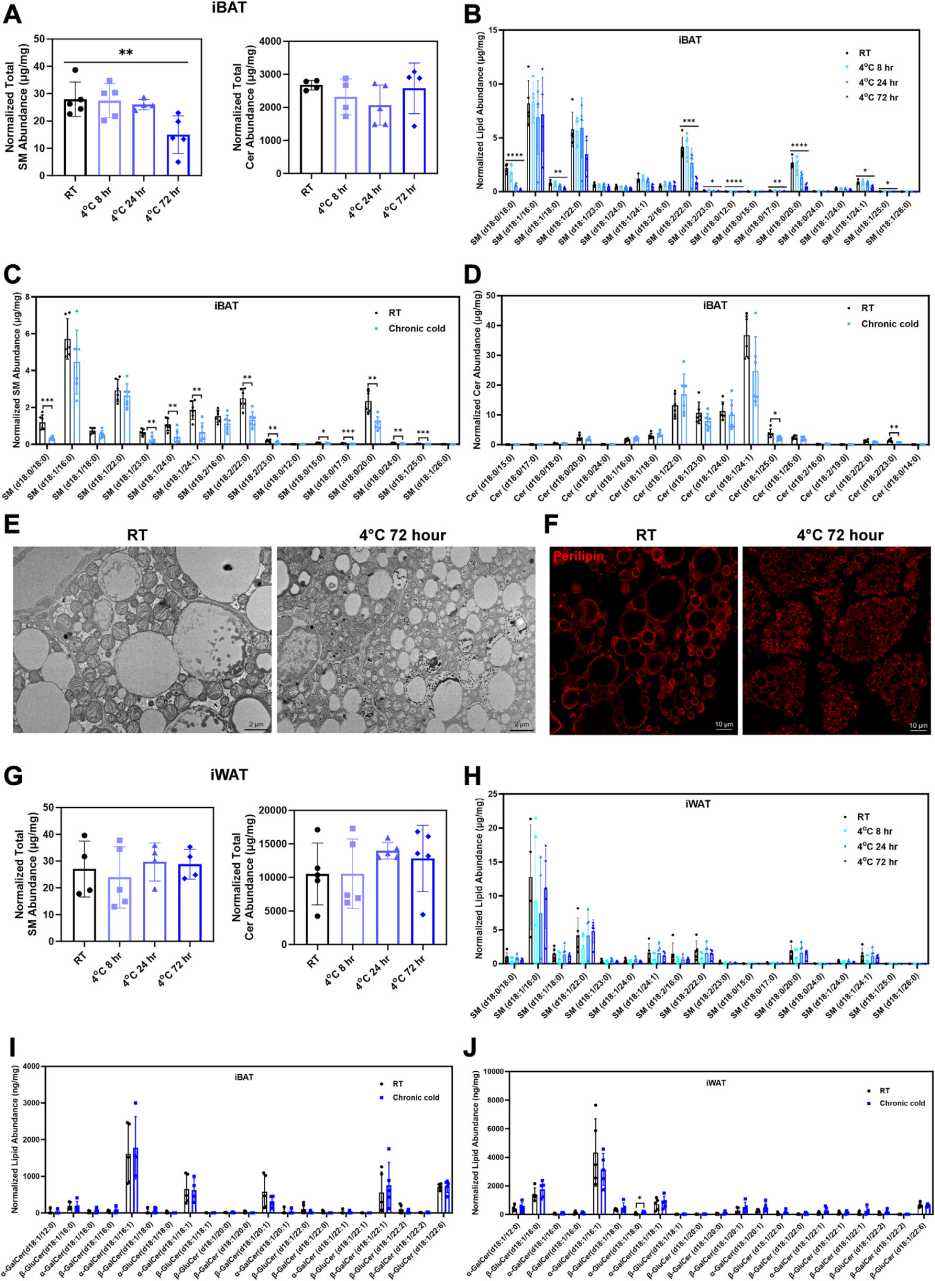
Sphingomyelin abundance in iBAT was negatively correlated with the length of cold exposure. A: Total abundance of sphingomyelin, but not ceramide, exhibited a significant decreasing trend as cold exposure duration increased. n = 4–5 animals per condition, represented by a dot in the histogram. ∗∗*P* < 0.01 by one-way ANOVA with Tukey’s posthoc test. B: Increasing number of sphingomyelin species in iBAT was found to be significantly reduced as the cold exposure proceeded. n = 4–5 animals per condition, represented by a dot in the histogram. ∗*P* < 0.05, ∗∗*P* < 0.01, ∗∗∗*P* < 0.001, ∗∗∗∗*P* < 0.0001 by one-way ANOVA with Tukey’s posthoc test. C: Most of the sphingomyelin species in iBAT remained at lower levels after chronic cold exposure (2 weeks at 18°C followed by 2 weeks at 10°C). n = 6 animals per condition, represented by a dot in the histogram. ∗*P* < 0.05, ∗∗*P* < 0.01, ∗∗∗*P* < 0.001, by unpaired *t* test with Welch's correction. D: Ceramide abundance in iBAT was relatively unaffected by chronic cold exposure. n = 6 animals per condition, represented by a dot in the histogram. ∗*P* < 0.05, ∗∗*P* < 0.01 by unpaired *t* test with Welch's correction. E, F: Transmission electron microscopy images (E) and perilipin immunostaining (F) of iBAT under room temperature (RT) and cold exposure (4°C for 72 h) conditions. Scale bar = 2 μm in (E) and 10 μm in (F). G, H:Total sphingomyelin and ceramide abundance (G) and individual species of sphingomyelin were unchanged in iWAT under different cold exposure conditions. n = 4–5 animals per condition, represented by a dot in the histogram. I, J: Individual galactosylceramide and glucosylceramide species were mostly unaffected in iBAT (I) and iWAT (J) under chronic cold condition. ∗*P* < 0.05 by unpaired *t* test with Welch's correction. Cer, ceramide; iBAT, interscapular brown adipose tissue; iWAT, inguinal white adipose tissue; LD, lipid droplet; Mt, mitochondria; SM, sphingomyelin.

To assess whether cold exposure affects the abundance of sphingomyelins, we performed targeted UPLC-HRMS on iBAT and iWAT from mice kept at either room temperature or 4°C for 8, 24, and 72 h. The total abundance of sphingomyelins, but not ceramide, was significantly decreased in iBAT as the animals entered nonshivering thermogenesis (>24 h) (Fig. 4A). Also, many individual sphingomyelin species exhibited a decreasing trend at this stage (Fig. 4B), which is well correlated with the increasing trend of UCP1 protein level (supplemental Fig. S1A–C). When exposing the animals to chronic cold condition (2 weeks at 18°C followed by 2 weeks at 10°C), the general trend in sphingomyelin (Fig. 4C) and ceramide (Fig. 4D) abundance remained the same. Transmission electron microscopy analysis of iBAT under room temperature and cold exposure condition revealed that the intracellular compartment of the adipocytes was mainly occupied by lipid droplets and mitochondria (Fig. 4E). Cold-exposed iBAT showed reduced lipid droplet size and increased mitochondria density (Fig. 4E). The reduction in lipid droplet size was further verified by Perilipin immunostaining (Fig. 4F). For iWAT, no changes were observed in sphingomyelin or ceramide abundance (Fig. 4G, H). In addition, most of the galactosylceramide and glucosylceramide species were not affected by chronic cold exposure in either iBAT or iWAT (Fig. 4I, J). These observations further suggest a specific role of sphingomyelin in iBAT during thermogenesis.

**Fig. 5:**
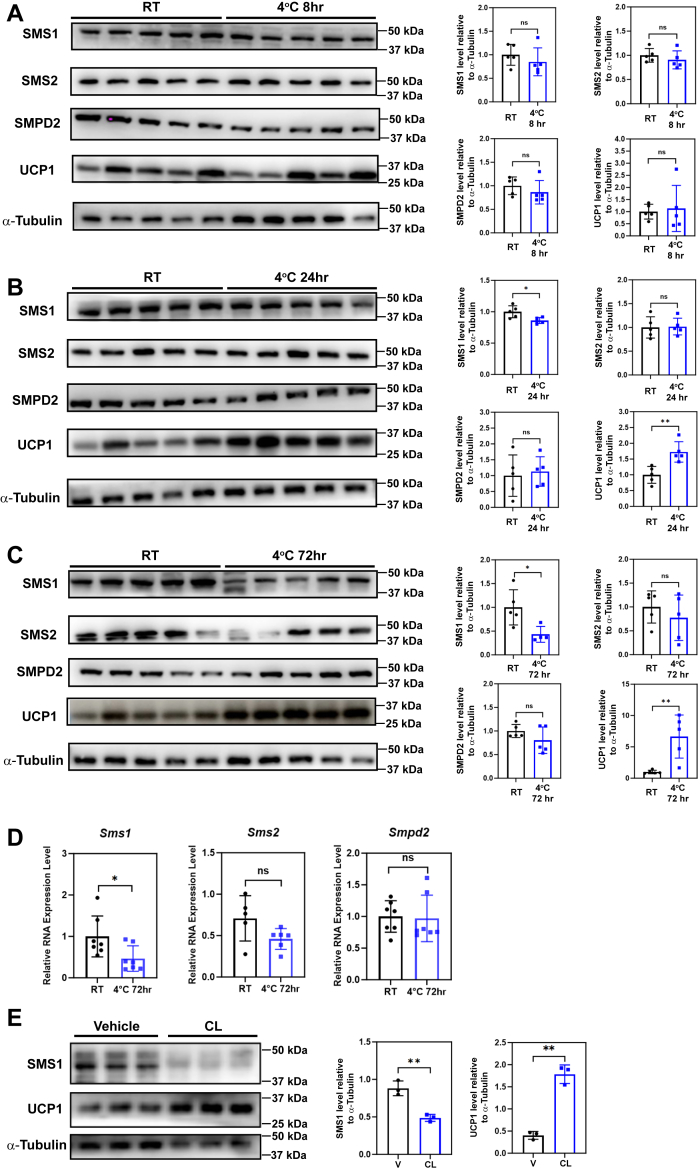
SMS1 protein levels in iBAT were reduced upon prolonged cold exposure. A–C: Protein level of SMS1, but not SMS2 or SMPD2, was significantly reduced in iBAT of animals that were exposed to cold condition for 24 (B) and 72 (C), but not 8 (A) hours. n = 5 animals per condition, represented by a dot in the quantification histogram. ∗*P* < 0.05, ∗∗*P* < 0.01 by unpaired *t* test with Welch's correction. D: mRNA level of *Sms1*, but not *Sms2* or *Smpd2*, was significantly reduced in iBAT of animals that were exposed to cold condition for 72 h. n = 5–7 animals per condition. ∗*P* < 0.05 by unpaired *t* test with Welch's correction. E: Protein level of SMS1 was significantly reduced in ex vivo-differentiated brown adipocytes treated with CL. n = 3 independent experiments. ∗∗*P* < 0.01 by unpaired *t* test with Welch's correction. hr, hour; iBAT, interscapular brown adipose tissue; ns, not significant; SMS, sphingomyelin synthase; SMPD, sphingomyelin phosphodiesterase; RT, room temperature.

Next, we investigated whether the reduced sphingomyelin abundance in iBAT was caused by alterations in the protein level of enzymes that are responsible for their synthesis and hydrolysis. Using the same experimental setup, we found that the level of SMS1, but not SMS2 or SMPD2, corelated well with sphingomyelin abundance (Fig. 5A–C). Consistently, the mRNA level of *Sms1*, but not *Sms2* or *Smpd2*, was significantly reduced after 72-h exposure to 4°C (Fig. 5D), suggesting that the cold-induced downregulation of SMS1 may occur at the transcription level. In agreement with the observation in vivo, a similar decrease in SMS1 protein level was observed in cultured brown adipocytes differentiated from primary iBAT SVF after treatment with a selective β3-adrenoceptor agonist, CL316, 243 (CL), which mimics the β-adrenergic stimulation that occurs upon cold exposure (Fig. 5E). These results suggest that cold exposure reduces the synthesis of sphingomyelin rather than its hydrolysis.

### Sphingomyelin content in iBAT negatively correlated with UCP1-mediated nonshivering thermogenesis and resistance to cold exposure

**Fig. 6:**
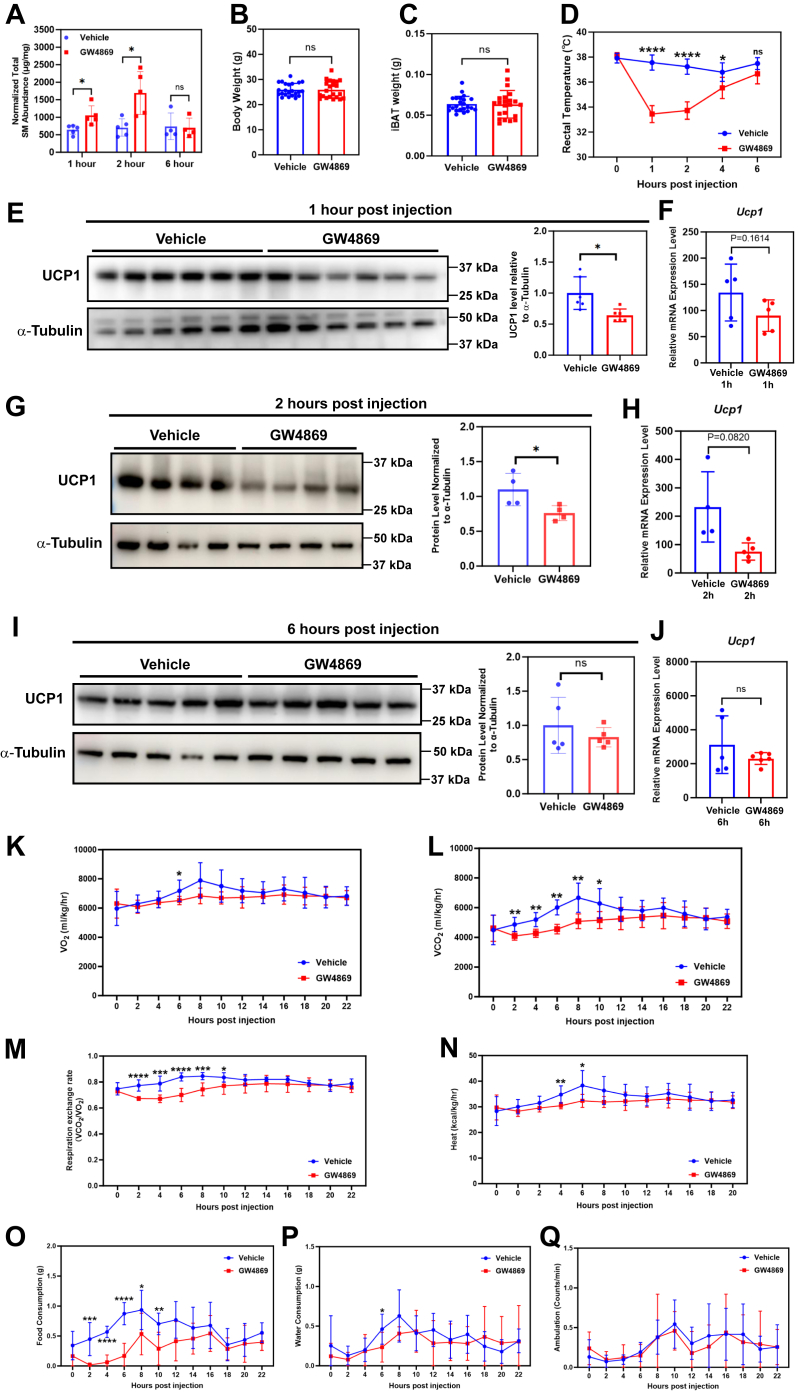
Increased sphingomyelin content reduced UCP1-mediated nonshivering thermogenesis in iBAT. A: Total sphingomyelin level in iBAT was significantly increased 1 and 2, but not 6 h after one injection of GW4869 in mice kept at 4°C. n = 4 or 5 animals per condition, represented by a dot in the histogram. ∗*P* < 0.05 by unpaired *t* test with Welch's correction. B, C: Body weight (B) and iBAT wet weight (C) were not affected by GW4869 injection. n = 22–23 animals per condition, represented by a dot in the histogram. D–J: Rectal temperature (D) and UCP1 protein level (E, G, and I) in iBAT were reduced at 1 and 2 h after GW4869 injection and recovered to comparable level at the 6-h time point. *Ucp1* mRNA level followed similar changing trend as the protein level, although no statistical significance was detected (F, H, and J). n = 5–12 animals per condition in (D). n = 4–6 animals per condition in (E–J), represented by a single band in the blot and a dot in the histogram. ∗*P* < 0.05, ∗∗∗∗*P* < 0.0001 by unpaired *t* test with Welch's correction. K–Q: Oxygen consumption (K), carbon dioxide release (L), respiration exchange rate (M), heat production (N), food (O) and water (P) consumption, and ambulation (Q) assessment of the vehicle and GW4869 injected animals. n = 7 animals per condition for (K–Q). ∗*P* < 0.05, ∗∗*P* < 0.01, ∗∗∗*P* < 0.001 by unpaired *t* test with Welch's correction. iBAT, interscapular brown adipose tissue; ns, not significant; UCP1, uncoupling protein 1.

Next, we assessed whether the reduction in sphingomyelin abundance observed after cold exposure has any impact on nonshivering thermogenesis and the maintenance of body temperature under cold temperatures. For this purpose, we injected 10-week-old mice that had been kept at 4°C for 72 h with GW4869, a selective neutral SMPD inhibitor (20), to prevent sphingomyelin hydrolysis in cold-exposed animals. GW4869 injection could significantly increase the total sphingomyelin level in iBAT at 1 and 2, but not 6 h after injection (Fig. 6A), without affecting the body weight or iBAT mass (Fig. 6B, C). In line with such trend, rectal temperature and UCP1 protein level in iBAT were both significantly reduced at the 1- and 2-h post GW4869 injection time points (Fig. 6D, E, and G), while both parameters became comparable at the 6-h time point (Fig. 6D, I). *Ucp1* mRNA level followed similar changing trend as the protein level, although statistical significance was not detected for any of the comparisons (Fig. 6F, H, and J). These results suggest that the diminished difference in rectal temperature at 6 h post GW4869 injection is likely due to the parallel recovery of the sphingomyelin content and UCP1 protein level in iBAT.

In term of metabolic profile, GW4869 injection majorly reduced the respiration exchange rate (RER) (Fig. 6M) as a result of decreased CO_2_ production (Fig. 6L). Oxygen consumption (Fig. 6K) and heat production (Fig. 6N) were relatively stable during the entire monitoring period, and the observed statistical significance is likely due to the variation in activity of the vehicle-injected animals during different time of the day. The RER values of both groups were below 0.9 (Fig. 6M), indicating that fat was likely to be employed as fuel source in addition to carbohydrate under cold exposure condition. Specifically, vehicle-injected animals had RER values around 0.8 at all time, indicating an approximate 1:2 ratio of carbohydrate and fat usage according to the manufacture’s guideline (Columbus). In contrast, the RER values of GW4869-injected animals during the first 10 h post injection were around 0.7, indicating almost 100% usage of fat as the energy source. In addition, these animals also had less food intake during this period (Fig. 6O), which may also account for their full dependence on the fat reservoir for energy supply. Of note, the observed alterations on core body temperature and food intake indicate that GW4869 injection has a systemic effect on thermogenesis and appetite that may arise from the effect of the chemical on other tissues. Water consumption (Fig. 6P) and ambulation (Fig. 6Q) were relatively unchanged. These results indicate that accumulation of sphingomyelin upon cold exposure might cause more rapid shift in substrate metabolism from carbohydrate to fat, which contributes to normal UCP1-mediated nonshivering thermogenesis and core body temperature maintenance. It is worth mentioning that the same experimental setup has been attempted under fasting condition; however, the animals could not cope with the cold exposure period without any food intake.

**Fig. 7:**
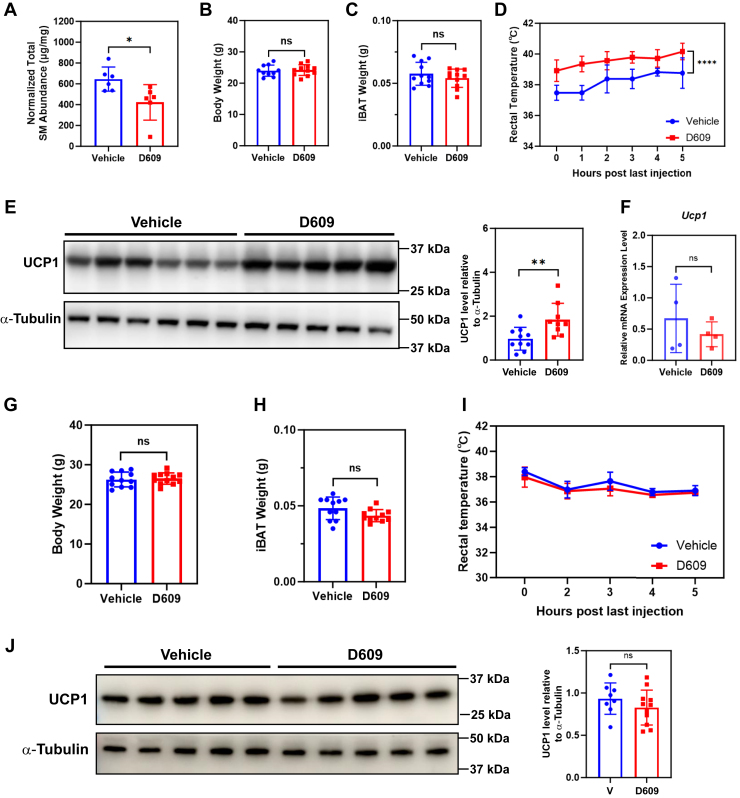
Reduced sphingomyelin content promoted UCP1-mediated thermogenesis. A: Total sphingomyelin level in iBAT was dramatically decreased after four consecutive daily injections of D609 in mice kept at 4°C. n = 6 animals per condition, represented by a dot in the histogram. ∗*P* < 0.05 by unpaired *t* test with Welch's correction. B, C: Body weight (B) and iBAT wet weight (C) were not affected by D609 injections. n = 10–11 animals per condition, represented by a dot in the histogram. D, E: Rectal temperature (D) and UCP1 protein level in iBAT (E) were increased in animals injected with D609. n = 5–6 animals per condition for (D), ∗∗∗∗*P* < 0.0001 by two-way ANOVA. n = 9–10 animals per condition for (E), ∗∗*P* < 0.01 by unpaired *t* test with Welch's correction. F: *Ucp1* mRNA level in iBAT was not affected by D609 injections. n = 4 animals per condition, represented by a dot in the histogram. ns, not significant by unpaired *t* test with Welch's correction. G–J: Four consecutive daily injections of D609 in mice kept at room temperature did not affect body weight (G), iBAT wet weight (H), rectal temperature (I), or UCP1 protein level in iBAT (J). n = 11 or 12 animals in (G, H), n = 8 or 11 animals in (J), represented by a dot in the histogram. n = 5–6 animals in (I). ns, not significant by unpaired *t* test with Welch's correction. iBAT, interscapular brown adipose tissue; UCP1, uncoupling protein 1.

Then, we investigated whether artificially decreasing sphingomyelin abundance in iBAT could enhance nonshivering thermogenesis during cold exposure. To this end, we placed 10-week-old mice at 4°C for 24 h prior to administration of 4 daily injections of D609, a potent inhibitor of both SMS1 and SMS2 (21), while the animals were kept at 4°C. After the last injection, sphingomyelin content was found to be significantly reduced in the iBAT of D609-injected animals (Fig. 7A). Body weight and iBAT mass remained comparable to control animals (Fig. 7B, C). In line with our hypothesis, we found that rectal temperature (Fig. 7D) and UCP1 protein level (Fig. 7E) in iBAT were both significantly increased after D609 injection. However, *Ucp1* mRNA level in iBAT did not show any obvious changing trend (Fig. 7F). Considering the nonsignificant changes in the *Ucp1* mRNA level in the iBAT of GW4869-injected animals (Fig. 6F, H, and J), it seemed that the regulation of UCP1 expression by sphingomyelin may be mainly at the translational level. Interestingly, D609 injection had no effect on mice kept at room temperature (Fig. 7G–J).

**Fig. 8:**
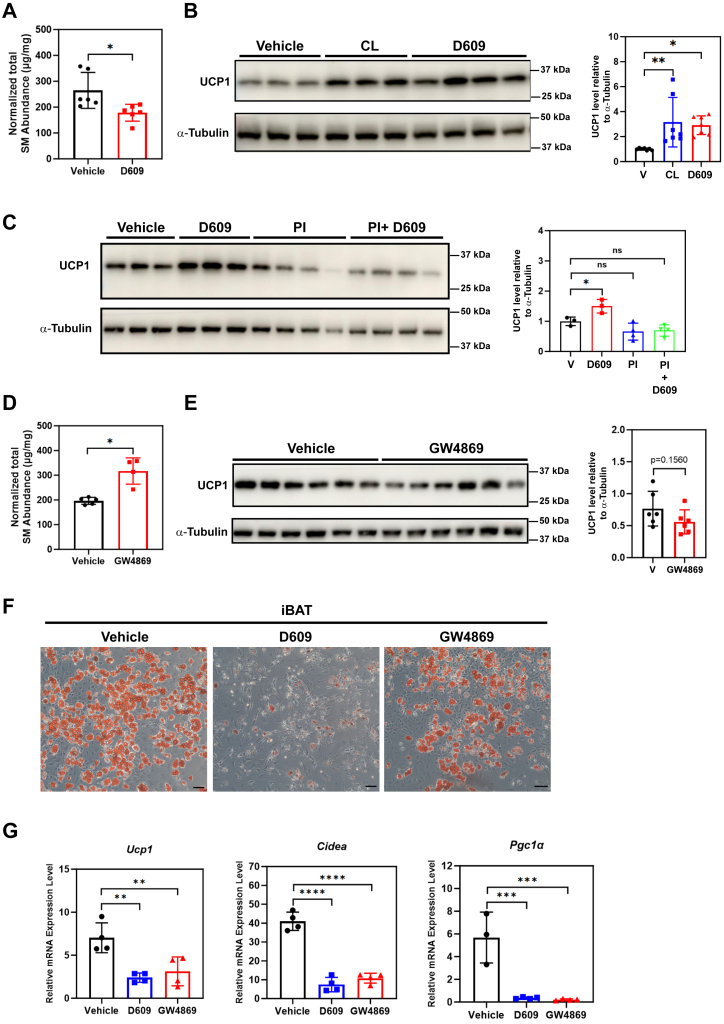
Reduced sphingomyelin content led to elevated UCP1 protein levels in ex vivo-differentiated brown adipocytes. A: 12-h treatment with D609 reduced the total sphingomyelin abundance in ex vivo-differentiated brown adipocytes. n = 6 wells from 2 independent experiments, represented by a dot in the graph. ∗*P* < 0.05 by unpaired *t* test with Welch's correction. B: UCP1 protein level was increased in D609-treated brown adipocytes. n = 7 wells of cells from two independent experiments, represented by a dot in the graph. ∗*P* < 0.05, ∗∗*P* < 0.01 by one-way ANOVA Tukey’s multiple comparisons test. C: Addition of protease inhibitor on top of D609 treatment reversed the sphingomyelin-induced increase in UCP1 protein level. n = 3–4 wells from two independent experiments, represented by a dot in the graph. ∗*P* < 0.05 by one-way ANOVA Tukey’s multiple comparisons test. D: 6-h treatment with GW4869 increased the total sphingomyelin abundance in fully differentiated brown adipocytes. n = 4–5 wells from two independent experiments, represented by a dot in the graph. ∗*P* < 0.05 by unpaired *t* test with Welch's correction. E: GW4869 treatment tended to reduce UCP1 protein level in ex vivo-differentiated brown adipocytes, although the difference did not reach statistical significance. n = 6 wells of cells from two independent experiments, represented by a dot in the graph. Statistical significance was assessed by unpaired *t* test with Welch's correction. F: Oil Red O staining images of ex vivo-differentiated adipocytes from iBAT that were treated with vehicle, D609 or GW4869 on DIV4. Scale bar = 100 μm. G: *Ucp1*, *Cidea*, and *Pgc1α* mRNA level was significantly reduced in ex vivo-differentiated brown adipocytes treated with D609 or GW4869 on DIV4. n = 3–4 wells of cells from two independent experiments, represented by a dot in the graph. ∗∗*P* < 0.01, ∗∗∗*P* < 0.001, ∗∗∗∗*P* < 0.0001 by one-way ANOVA Tukey’s multiple comparisons test. DIV, days in vitro; iBAT, interscapular brown adipose tissue; PI, protease inhibitor; UCP1, uncoupling protein 1; V, vehicle.

D609 treatment of ex vivo-differentiated iBAT adipocytes also reduced total sphingomyelin content (Fig. 8A) and led to significant increase in UCP1 protein level, comparable to those attained following CL treatment (Fig. 8B). To check the impact of protease inhibitor on the sphingomyelin-induced UCP1 protein level change, we treated fully differentiated iBAT adipocytes with a cocktail of protease inhibitors and found that adding protease inhibitors on top of the D609 treatment abolished the elevated UCP1 protein level induced by D609 treatment alone (Fig. 8C), implicating a role of protein degradation in sphingomyelin-induced UCP1 level change. Conversely, treatment of cultured brown adipocytes with GW4869 significantly increased total sphingomyelin content (Fig. 8D) and had a tendency to reduce the UCP1 protein level (Fig. 8E), although statistical significance was not reached. To check whether alteration in sphingomyelin level is required for ex vivo differentiation of brown adipocytes, we transiently applied D609 or GW4869 to primary cultures of iBAT SVF cells on DIV4 and collected on DIV9 for analysis. We found that Oil Red O-stained adipocytes were almost completely lost in D609-treated cells (Fig. 8F). GW4869-treated cells also showed reduced differentiation efficiency (Fig. 8F). In addition, *Ucp1*, *Cidea*, and *Pgc1α* mRNA levels were all significantly reduced in cultures treated with either chemical (Fig. 8G). These results suggest that a proper level of sphingomyelin is needed to promote the differentiation of iBAT SVF cells into brown adipocytes.

Taken together, these results revealed a negative correlation between sphingomyelin abundance and nonshivering thermogenic capacity.

## Discussion

Alterations in adipocyte lipid composition not only affect energy supply but also modulate physiological functions in adipose tissue and elsewhere in the body. It has been speculated that a substantial difference in lipid composition exists between ASPCs and adipocytes, and a previous study comparing the lipid profiles of different mesenchymal precursor-, preadipocyte- and brown adipose-derived cell line models supports such notion (22). In the present study, we investigated lipidomic dynamics during adipogenesis and differentiation of both brown and white adipocytes. In addition to the expected accumulation of glycerolipids that are responsible for energy storage, we found that sphingomyelins, but not other sphingolipid species, were specifically enriched in ex vivo-differentiated brown adipocytes, as well as mature adipocytes freshly isolated from iBAT. Sphingomyelin is a bioactive lipid that is believed to mediate cellular signaling via molecules derived from its metabolism, as well as sphingomyelin-enriched lipid rafts located in the plasma membrane (13). Its functional role has been implicated in many biological contexts, including inflammatory signaling, cholesterol homeostasis, and insulin sensitivity. In adipose tissue, loss of the major sphingomyelin-producing enzyme SMS1 led to WAT atrophy as a result of elevated oxidative stress (23). Moreover, SMS2-deficient mice are resistant to high-fat-diet-induced obesity and insulin resistance (24) and display elevated energy expenditure in subcutaneous WAT and reduced expression of genes involved in fatty acid synthesis in BAT (25). Here, we showed that inhibition of SMS function in animals exposed to cold temperature could enhance UCP1-mediated nonshivering thermogenesis, while animals with compromised SMPD function failed to maintain body temperature under those conditions. Blocking sphingomyelin synthesis in ex vivo-differentiated brown adipocytes led to elevated UCP1 protein level. These results suggest that sphingomyelins may act as a clutch to maintain brown adipocytes at a resting state in the absence of environmental stimulation. However, UCP1 levels of animals kept at room temperature did not change upon D609 injection, which would indicate more complex roles in vivo. Indeed, under cold exposure, decreased sphingomyelin content was reciprocally correlated to the increased UCP1 protein levels in iBAT.

We hypothesize that reduced sphingomyelin content in iBAT after cold exposure is caused by a reduction in SMS1 protein levels. So far, not much is known about the mechanisms that regulate SMS1 expression. At the protein level, SMS1 is known to be cleaved by caspases as a result of Fas ligand-triggered cell death, leading to its release from the Golgi complex into the cytoplasm (26). At the mRNA level, multiple alternatively spliced transcripts of SMS1 with varying exon combinations and 5’-UTR and 3’-UTR lengths have been found in adult human tissues (27). Expression of these transcripts is regulated by tissue-specific polyadenylation at intronic sites, resulting in truncated transcripts that do not contribute to full-length SMS1 protein synthesis (28). Circular noncoding RNAs containing 5’-UTR, exon, or intron sequences of the SMS1 gene have been found in human, rat, and mouse brain tissues and are predicted to interact with SMS1 mRNA as well as Ago proteins (29). A previous study demonstrated the role of microRNA-33 in the maintenance of adaptive thermogenesis during cold stress by enhancing sympathetic nerve activity (30). Thus, microRNAs may mediate suppression of SMS1 expression during cold exposure. Recently, SMPD3 has been shown to be a direct phosphorylation target of AMPK, contributing to the progression of nonalcoholic fatty liver disease to nonalcoholic steatohepatitis (31). This neutral sphingomyelinase has also been implicated in the context of atherosclerotic progression as a direct target of HIF-1α (32). Some of these regulatory mechanisms of SMPD3 may underlie the effects of GW4869 in animals exposed to cold.

A previous study has shown that cold exposure lowered the total ceramide content in visceral and subcutaneous WAT, without affecting the sphingomyelin levels (33). We did not observe any changes in ceramide, galactosylceramide, or glucosylceramide abundance in the iBAT of cold-exposed animals, in line with the distinct roles of sphingomyelin and ceramide in BAT and WAT. We speculate that the effects of sphingomyelin in iBAT are likely attributed to its abundance in membrane-associated lipid rafts, rather than due to its metabolic derivatives. The overall changes in sphingomyelin abundance upon cold exposure could be caused by organelle-specific alterations. Changes of sphingomyelin content in the inner membrane of mitochondria may alter the membrane fluidity, leading to UCP1 release to the cytoplasm and subsequent degradation. Previous studies have demonstrated several UCP1 regulatory mechanisms, including allosteric association with free fatty acids (34), sulfenylation of Cys^253^ in response to reactive oxygen species (35), and Sirt5-mediated succinylation that reduced protein stability and function (36). Any of these processes could also be involved in sphingomyelin-mediated UCP1 depletion.

Most of the abundant sphingomyelin species demonstrated a decreasing trend upon cold exposure, except for SM (d18:1/16:0), despite of its enrichment in the brown adipocytes. In addition, abundance of the other C16 sphingomyelin, SM (d18:2/16:0) identified in the targeted sphingomyelin dataset was also not affected by cold, suggesting that sphingomyelins with C16 acyl chain length might be specifically resistant to cold exposure. One possible explanation is the subcellular localization of the C16 sphingomyelins, that is, the C16 sphingomyelins are specifically located in certain organelle(s) that are less affected upon cold exposure. In line with this notion, SMS1 has been shown to mainly reside in the Golgi, while SMS2 is primarily localized to plasma membrane (37). Our observation that cold exposure only affected SMS1 protein level, but not SMS2, suggests that the changes in specific sphingomyelin species upon cold exposure may be attributed to their specific subcellular localization. During ex vivo brown adipocyte adipogenesis, the identified C16 sphingomyelin SM 34:0,O2 remained at a relatively high level at all three tested stages (data not shown), suggesting that it might be an essential component for the entire adipogenic process and therefore is more resistant to environmental changes. In line with this, pattern 1 sphingomyelin, SM 38:0,O2 (estimated as SM d18:0/20:0 in the targeted sphingolipid dataset based on carbon chain length and number of double-bonds) that showed increasing trend in abundance during adipogenesis was greatly reduced upon cold exposure.

In summary, our results demonstrate the specific accumulation of sphingomyelins during maturation of BAT adipocytes. Upon cold exposure, sphingomyelin abundance decreases concomitantly with reduced SMS1 expression, which we propose is a necessary step for the maintenance of nonshivering thermogenesis in BAT. One limitation of the present study is that the D609 chemical is not an SMS-specific inhibitor. It also inhibits phosphatidylcholine-specific phospholipase C that impinges on the lipid second messengers 1,2-diacylglycerol. Therefore, manipulation of sphingomyelin level with genetic approaches is needed to confirm its specific role in thermogenesis. Moreover, intraperitoneal injection of GW4869 likely had systemic effects that arose from other tissues in addition to iBAT, reflected by changes in core temperature and food intake. Local injection of GW4869 to iBAT is needed to further explore the role of sphingomyelin in thermogenic capacity of brown adipocytes. Furthermore, other factors that affect thermogenesis and body temperature regulation need to be tested for a more comprehensive view on the role of sphingomyelin content in thermogenic capacity. Future work should be dedicated to further characterize the role of sphingomyelin in transgenic mouse models lacking sphingomyelin synthases and hydrolases in brown adipocytes.

## Data availability

All data are contained within the manuscript.

## Supplemental data

This article contains supplemental data.

## Conflict of interest

The authors declare that they have no conflicts of interest with the contents of this article.

## Supplemental data

Supplemental Fig. S1 UCP1 protein and mRNA levels were elevated after prolonged cold exposure. A–C, E and F: Western blots and quantifications of UCP1 protein level changes in iBAT (A–C) and iWAT (E, F) during different cold exposure periods. n = 3–5 animals per condition, represented by a dot in the quantification histogram. ∗∗*P* < 0.01 by unpaired t test with Welch's correction. ns, not significant. RT, room temperature. hr, hour. D: *Ucp1* mRNA level in iBAT during different cold exposure periods. n = 4 or 7 animals per condition. ∗∗ *P* < 0.01, ∗∗∗∗ *P* < 0.0001 by unpaired t test with Welch's correction.

## References

[1] Cannon B., Nedergaard J. (2004). Brown adipose tissue: function and physiological significance. Physiol. Rev..

[2] Enerbäck S., Jacobsson A., Simpson E.M., Guerra C., Yamashita H., Harper M.E. (1997). Mice lacking mitochondrial uncoupling protein are cold-sensitive but not obese. Nature.

[3] Fedorenko A., Lishko P.V., Kirichok Y. (2012). Mechanism of fatty-acid-dependent UCP1 uncoupling in brown fat mitochondria. Cell.

[4] Cao W., Medvedev A.V., Daniel K.W., Collins S. (2001). beta-Adrenergic activation of p38 MAP kinase in adipocytes: cAMP induction of the uncoupling protein 1 (UCP1) gene requires p38 MAP kinase. J. Biol. Chem..

[5] Petrovic N., Walden T.B., Shabalina I.G., Timmons J.A., Cannon B., Nedergaard J. (2010). Chronic peroxisome proliferator-activated receptor gamma (PPARgamma) activation of epididymally derived white adipocyte cultures reveals a population of thermogenically competent, UCP1-containing adipocytes molecularly distinct from classic brown adipocytes. J. Biol. Chem..

[6] Wu J., Bostrom P., Sparks L.M., Ye L., Choi J.H., Giang A.H. (2012). Beige adipocytes are a distinct type of thermogenic fat cell in mouse and human. Cell.

[7] Fahy E., Subramaniam S., Murphy R.C., Nishijima M., Raetz C.R., Shimizu T. (2009). Update of the LIPID MAPS comprehensive classification system for lipids. J. Lipid Res..

[8] Muro E., Atilla-Gokcumen G.E., Eggert U.S. (2014). Lipids in cell biology: how can we understand them better?. Mol. Biol. Cell.

[9] Schweizer S., Liebisch G., Oeckl J., Hoering M., Seeliger C., Schiebel C. (2019). The lipidome of primary murine white, brite, and brown adipocytes-Impact of beta-adrenergic stimulation. PLoS Biol..

[10] Marcher A.B., Loft A., Nielsen R., Vihervaara T., Madsen J.G., Sysi-Aho M. (2015). RNA-seq and mass-spectrometry-based lipidomics reveal Extensive changes of glycerolipid pathways in Brown adipose tissue in response to cold. Cell Rep..

[11] Merrill A.H.J. (2011). Sphingolipid and glycosphingolipid metabolic pathways in the era of sphingolipidomics. Chem. Rev..

[12] Merrill A.H., Wang M.D., Park M., Sullards M.C. (2007). (Glyco)sphingolipidology: an amazing challenge and opportunity for systems biology. Trends Biochem. Sci..

[13] Chakraborty M., Jiang X.C. (2013). Sphingomyelin and its role in cellular signaling. Adv. Exp. Med. Biol..

[14] Muralidharan S., Shimobayashi M., Ji S., Burla B., Hall M.N., Wenk M.R. (2021). A reference map of sphingolipids in murine tissues. Cell Rep..

[15] Ullman M.D., Radin N.S. (1974). The enzymatic formation of sphingomyelin from ceramide and lecithin in mouse liver. J. Biol. Chem..

[16] Tafesse F.G., Ternes P., Holthuis J.C. (2006). The multigenic sphingomyelin synthase family. J. Biol. Chem..

[17] Pavoine C., Pecker F. (2009). Sphingomyelinases: their regulation and roles in cardiovascular pathophysiology. Cardiovasc. Res..

[18] Pang Z., Chong J., Li S., Xia J. (2020). MetaboAnalystR 3.0: toward an optimized workflow for global metabolomics. Metabolites.

[19] Nedergaard J., Golozoubova V., Matthias A., Asadi A., Jacobsson A., Cannon B. (2001). UCP1: the only protein able to mediate adaptive non-shivering thermogenesis and metabolic inefficiency. Biochim. Biophys. Acta.

[20] Luberto C., Hassler D.F., Signorelli P., Okamoto Y., Sawai H., Boros E. (2002). Inhibition of tumor necrosis factor-induced cell death in MCF7 by a novel inhibitor of neutral sphingomyelinase. J. Biol. Chem..

[21] Luberto C., Hannun Y.A. (1998). Sphingomyelin synthase, a potential regulator of intracellular levels of ceramide and diacylglycerol during SV40 transformation. Does sphingomyelin synthase account for the putative phosphatidylcholine-specific phospholipase C?. J. Biol. Chem..

[22] Liaw L., Prudovsky I., Koza R.A., Anunciado-Koza R.V., Siviski M.E., Lindner V. (2016). Lipid profiling of in vitro cell models of adipogenic differentiation: relationships with mouse adipose tissues. J. Cell Biochem..

[23] Yano M., Yamamoto T., Nishimura N., Gotoh T., Watanabe K., Ikeda K. (2013). Increased oxidative stress impairs adipose tissue function in sphingomyelin synthase 1 null mice. PLoS One.

[24] Mitsutake S., Zama K., Yokota H., Yoshida T., Tanaka M., Mitsui M. (2011). Dynamic modification of sphingomyelin in lipid microdomains controls development of obesity, fatty liver, and type 2 diabetes. J. Biol. Chem..

[25] Hanamatsu HM S., Sakai S., Okazaki T., Watanabe K., Igarashi Y., Yuyama K. (2018). Multiple roles of Sms2 in white and Brown adipose tissues from dietinduced obese mice. J. Metab. Synd.

[26] Lafont E., Milhas D., Carpentier S., Garcia V., Jin Z.X., Umehara H. (2010). Caspase-mediated inhibition of sphingomyelin synthesis is involved in FasL-triggered cell death. Cell Death Differ..

[27] Rozhkova A.V., Dmitrieva V.G., Zhapparova O.N., Sudarkina O.Y., Nadezhdina E.S., Limborska S.A. (2011). Human sphingomyelin synthase 1 gene (SMS1): organization, multiple mRNA splice variants and expression in adult tissues. Gene.

[28] Dergunova L.V., Rozhkova A.V., Sudarkina O.Y., Limborska S.A. (2013). The use of alternative polyadenylation in the tissue-specific regulation of human SMS1 gene expression. Mol. Biol. Rep..

[29] Filippenkov I.B., Sudarkina O.Y., Limborska S.A., Dergunova L.V. (2015). Circular RNA of the human sphingomyelin synthase 1 gene: multiple splice variants, evolutionary conservatism and expression in different tissues. RNA Biol..

[30] Horie T., Nakao T., Miyasaka Y., Nishino T., Matsumura S., Nakazeki F. (2021). microRNA-33 maintains adaptive thermogenesis via enhanced sympathetic nerve activity. Nat. Commun..

[31] Chen B., Sun L., Zeng G., Shen Z., Wang K., Yin L. (2022). Gut bacteria alleviate smoking-related NASH by degrading gut nicotine. Nature.

[32] Wang P., Zeng G., Y Y., Zhang S.Y., Dong Y., Zhang Y. (2022). Disruption of adipocyte HIF-1 α improves atherosclerosis through the inhibition of ceramide generation. Acta Pharm. Sin. B.

[33] Chaurasia B., Kaddai V.A., Lancaster G.I., Henstridge D.C., Sriram S., Galam D.L. (2016). Adipocyte ceramides regulate subcutaneous adipose browning, inflammation, and metabolism. Cell Metab..

[34] Divakaruni A.S., Humphrey D.M., Brand M.D. (2012). Fatty acids change the conformation of uncoupling protein 1 (UCP1). J. Biol. Chem..

[35] Chouchani E.T., Kazak L., Jedrychowski M.P., Lu G.Z., Erickson B.K., Szpyt J. (2016). Mitochondrial ROS regulate thermogenic energy expenditure and sulfenylation of UCP1. Nature.

[36] Wang G., Meyer J.G., Cai W., Softic S., Li M.E., Verdin E. (2019). Regulation of UCP1 and mitochondrial metabolism in Brown adipose tissue by reversible succinylation. Mol. Cell.

[37] Huitema K., van den Dikkenberg J., Brouwers J.F., Holthuis J.C. (2004). Identification of a family of animal sphingomyelin synthases. EMBO J..

