# Supplementary figures and images for: Sphingomyelin is involved in regulating UCP1-mediated nonshivering thermogenesis
Source: J Lipid Res. 2024 May 9;65(6):100559. doi: 10.1016/j.jlr.2024.100559 (PMC11166878; doi:10.1016/j.jlr.2024.100559)

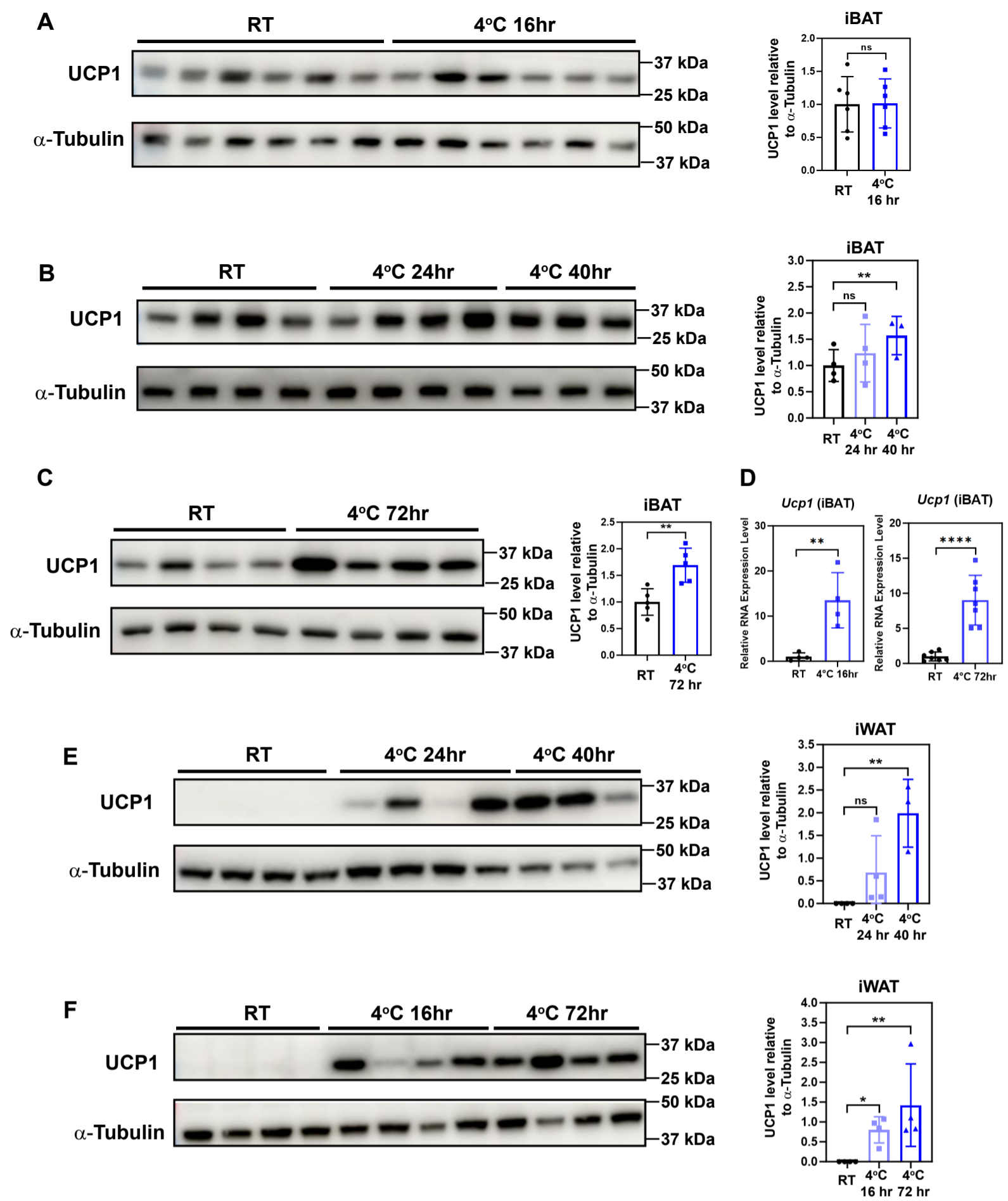

Supplement: Supplemental Fig. S1 — UCP1 protein and mRNA levels were elevated after prolonged cold exposure. A–C, E and F: Western blots and quantifications of UCP1 protein level changes in iBAT (A–C) and iWAT (E, F) during different cold exposure periods. n = 3–5 animals per condition, represented by a dot in the quantification histogram. ∗∗P < 0.01 by unpaired t test with Welch's correction. ns, not significant. RT, room temperature. hr, hour. D: Ucp1 mRNA level in iBAT during different cold exposure periods. n = 4 or 7 animals per condition. ∗∗ P < 0.01, ∗∗∗∗ P < 0.0001 by unpaired t test with Welch's correction. [file mmc1.pdf]
